# A phage displaying an Aβ-interacting peptide mitigates neurotoxicity and prevents Aβ-driven gene expression changes

**DOI:** 10.3389/fnmol.2025.1716626

**Published:** 2025-12-10

**Authors:** Laura Maria De Plano, Luigi Chiricosta, Simone D’Angiolini, Alessandra Saitta, Alessandra Trainito, Serena Silvestro, Sabrina Conoci, Salvatore Oddo, Antonella Caccamo

**Affiliations:** 1 Department of Chemical, Biological, Pharmaceutical and Environmental Sciences, University of Messina, Messina, Italy; 2 IRCCS Centro Neurolesi "Bonino-Pulejo", Messina, Italy; 3 Department of Chemistry, Biology and Biotechnology, Perugia, Italy

**Keywords:** Alzheimer’s disease, amyloid-beta, phage display, neuroprotection, gene expression profiling

## Abstract

**Introduction:**

Alzheimer’s disease (AD) is characterized by the accumulation of amyloid-beta (Aβ) peptides, which contribute to synaptic dysfunction, neuronal toxicity, and gene expression alterations. In a previous study, we identified a phage displaying a peptide that selectively interacts with Aβ autoantibodies.

**Methods:**

Here, we assessed whether this phage also directly interacts with Aβ, as predicted through bioinformatic analyses. We evaluated its functional effects in a neuronal cell line exposed to Aβ and performed transcriptomic profiling by RNA sequencing.

**Results:**

We demonstrate that the phage directly interacts with Aβ, consistent with bioinformatic predictions. Functionally, the phage protected the neuronal cell line from Aβ-induced toxicity. RNA sequencing revealed that the phage prevented Aβ-induced alterations in the expression of 1,819 genes, suggesting a role in modulating Aβ-associated metabolic changes.

**Discussion:**

These findings highlight the therapeutic potential of phage-displayed peptides in counteracting Aβ toxicity and restoring cellular homeostasis, laying a foundation for future investigations into phage-based interventions for AD.

## 1 Introduction

Alzheimer’s disease (AD) is the most common neurodegenerative disorder, characterized by progressive cognitive decline and memory impairment (Querfurth and LaFerla, 2010; Scheltens et al., 2021). Clinically, AD manifests with symptoms ranging from mild forgetfulness in early stages to severe dementia and loss of independence in later stages (Querfurth and LaFerla, 2010; Scheltens et al., 2021). Pathologically, AD is characterized by the accumulation of extracellular amyloid-beta (Aβ) plaques and intracellular neurofibrillary tangles composed of hyperphosphorylated tau protein (Querfurth and LaFerla, 2010; Scheltens et al., 2021). Additionally, AD is associated with synaptic dysfunction, neuroinflammation, and widespread neuronal loss, particularly in the hippocampus and cortex (Querfurth and LaFerla, 2010; Taddei and Karen, 2025). Aβ oligomers, rather than monomeric forms, are now recognized as the principal neurotoxic species, as they disrupt synaptic function, induce oxidative and metabolic stress, and correlate more strongly with cognitive decline than amyloid plaque burden (Broersen et al., 2010; Goure et al., 2014). Despite recent advances in the field, the molecular mechanisms underlying Aβ-induced toxicity remain elusive.

Genomic studies conducted on various cohorts by independent laboratories have revealed widespread gene expression changes in AD. These transcriptomic analyses have identified dysregulated pathways involved in neuroinflammation, synaptic function, and cellular metabolism, providing new insights into the molecular mechanisms contributing to AD pathology (He et al., 2024; Aguzzoli Heberle et al., 2025; Liu and Song, 2025). Such studies underscore the complexity of AD at the gene regulatory level and highlight potential new targets for therapeutic intervention (Aguzzoli Heberle et al., 2025).

Currently, disease-modifying therapies for AD are still limited, and existing therapies provide only symptomatic relief without modifying disease progression (Thawabteh et al., 2024; Soni et al., 2025). The recent development of monoclonal antibodies targeting Aβ aggregates, such as aducanumab and lecanemab, has shown promise in reducing the amyloid burden (Aljuhani et al., 2024; Chhabra et al., 2024). However, these therapies have been met with significant criticism due to their limited clinical efficacy and associated risks, including amyloid-related imaging abnormalities (Doran and Sawyer, 2024; Heidebrink and Paulson, 2024). Thus, there is a pressing need for novel and innovative therapeutic approaches that effectively target the pathological mechanisms of AD while minimizing risks.

Phage display technology represents a powerful and versatile tool for identifying peptides and antibodies with high specificity for disease-associated targets (Rizzo et al., 2023; Jahandar-Lashaki et al., 2024). This technique involves the presentation of peptide or antibody libraries on the surface of bacteriophages, allowing for the selection of molecules that interact with specific proteins of interest (Smith, 1985). In the context of neurodegenerative diseases, phage display has been utilized to identify peptides and antibodies that selectively bind to misfolded proteins, including Aβ and tau, offering potential therapeutic and diagnostic applications (Droste et al., 2015; Fassler et al., 2022; Rizzo et al., 2023; Nair et al., 2024). Given its capacity to generate highly specific binding molecules, phage display provides a promising platform for the development of novel AD interventions aimed at mitigating Aβ-induced toxicity.

Recently, we identified phages that recognized circulating Aβ autoantibodies in the sera of AD patients and could discriminate between healthy controls and AD patients with a high degree of accuracy (Rizzo et al., 2023). Here, we show that one of these phages, namely 12CIII1, recognizes Aβ and prevents its toxicity *in vitro* by reverting many of the Aβ-induced changes in gene expression.

## 2 Materials and methods

### 2.1 Computational analysis

The Protein Data Bank (PDB) 12CIII1-pVIII protein structure model was built using MODELLER 9.20, as previously reported (Williams, 1988). Briefly, the structure of the pVIII protein was obtained from the RCSB PDB (PDB ID: 2mjz). The amino acid sequence of the engineered pVIII protein from the 12CIII1 clone was formatted in FASTA format to generate the pVIII-engineered model: 1-AEGEFGGGCIEGPCLEGDPAKAAFNSLQASATEYIGYAWAMVVVIVGATIGIKLFKKFTSKAS-64 (the sequence of the foreign peptide is shown in bold). The best Three-Dimensional (3D) model was selected based on the lowest Discrete Optimized Protein Energy (DOPE) value, which indicates the optimal construction energy.

The exposed 12CIII1-pVIII protein was analyzed for its potential binding to various assembly states of Aβ (ranging from 1 to 12 monomers). The 3D structure of Aβ was obtained from the RCSB PDB (PDB ID: 2NAO)^1^. The 2NAO 3D structure was remodeled using YASARA software to generate 3D structures of Aβ containing 1, 3, 6, 9, and 12 monomers (labeled with letters from A through N). The 12CIII1-pVIII protein structure was used as input 1 in ZDOCK^2^, while the remodeled Aβ structures (with varying monomer counts) were used as input 2 (Pierce et al., 2014).

Docking was performed by specifying the binding residues on the exposed region of the 12CIII1-pVIII protein (amino acid positions 1–40) to obtain docking results that best reflect the most relevant interaction patterns. The program output provided docking predictions for the 12CIII1-pVIII protein with each Aβ assembly state, filtered based on the ZDOCK score. The PDB files of the top three docking models for each docking set were analyzed using iCn3D^3^ to evaluate interaction features, including hydrogen bonding (threshold: 3.8 Å), salt bridges (threshold: 6 Å), and *π*-stacking interactions (threshold: 6 Å) (Wang et al., 2020).

The binding free energies (ΔG, kcal/mol) of the docked complexes were estimated using the PRODIGY web server (Xue et al., 2016). PRODIGY predicts the binding affinity based on interfacial contacts and non-interacting surface properties derived from the protein–protein complex structure. More negative ΔG values indicate stronger and more stable interactions.

### 2.2 Cell culture and differentiation

Human neuroblastoma cell line SH-SY5Y were cultured in Dulbecco’s Modified Eagle Medium/Nutrient Mixture F-12 (DMEM/F-12; Thermo Fisher Scientific, Carlsbad, CA, USA) supplemented with 15% fetal bovine serum (FBS; Sigma-Aldrich, Saint Louis, MO, USA) and 1% penicillin–streptomycin (Thermo Fisher Scientific, Carlsbad, CA, USA). Cells were maintained at 37 °C in a humidified incubator with 5% CO₂. For differentiation, we modified a protocol described previously (de Medeiros et al., 2019). Specifically, SH-SY5Y cells were seeded at an appropriate density in culture plates and allowed to adhere overnight in a growth medium containing 15% FBS. The following day, the medium was replaced with a differentiation medium containing 2.5% FBS and 10 μM retinoic acid (RA; Sigma-Aldrich, Saint Louis, MO, USA). Every 3 days, the medium was changed with progressively lower concentrations of FBS as follows: 1% FBS with 10 μM RA, followed by two consecutive medium changes containing 0.5% FBS with 10 μM RA. Differentiation was carried out for a total of 13 days. At the end of the differentiation process, cells were treated for 24 h with 2 μM Aβ42 or with 1,011 Transducing Units per milliliter (TU/ml) 12CIII1, which was added 1 h prior to Aβ treatment.

### 2.3 MTT assay

For the MTT (3-(4,5-dimethylthiazol-2-yl)-2,5-diphenyltetrazolium bromide) assay, 7,000 cells per well were plated in a 96-well plate (n = 8 wells per treatment). Following treatment, an MTT assay was performed to assess cell viability. MTT solution (5 mg/mL, Sigma-Aldrich) was added to each well and incubated for 2.5 h, allowing metabolically active cells to reduce it to purple formazan crystals. After incubation, the MTT solution was removed, and the formazan crystals were dissolved in 100 μL of Dimethyl Sulfoxide (DMSO; Sigma-Aldrich). Absorbance was measured at 540 nm, with the signal intensity directly proportional to the amount of formazan formed, reflecting cell viability. Absorbance values from the control group were used as a baseline to calculate the percent viability of the treated groups. Although LDH release assays can provide complementary information on membrane integrity, the MTT assay remains a robust and widely used indicator of neuronal viability, particularly under conditions of Aβ-induced metabolic stress, where mitochondrial dysfunction precedes overt cell death.

### 2.4 Neurite length

To measure neurite length, 20,000 cells per well were plated in a 24-well plate, with eight wells per treatment group (*n* = 8). Three images were captured from each well to ensure accurate measurements. Neurite lengths were then quantified using ImageJ software with the SNT (Sholl Analysis Tool) extension (Binley et al., 2014). ImageJ automatically detected the image size in pixels, and neurite length measurements were based on these pixel counts. To convert pixel measurements to μm, a conversion factor of 1 μm = 4.287525 pixels was applied.

### 2.5 RNA extraction and library preparation

SH-SY5Y cells were seeded in 6-well plates at a density of 1.5 × 10^5^ cells per well in 2 mL of DMEM/F-12 (see Section 2.2) and then differentiated. After 13 days of retinoic acid-induced differentiation, SH-SY5Y cells were treated with Aβ42 for 24 h, either in the presence or absence of 12CIII1. Following treatment, cell pellets were collected, and total RNA was extracted using the Maxwell® RSC instrument according to the manufacturer’s instructions for the Maxwell® RSC simplyRNA Cells Kit (Promega, Madison, WI, USA).

Complementary DNA (cDNA) library preparation was performed on two biological replicates for each of the three conditions using the TruSeq® RNA Exome protocol (Illumina, San Diego, CA, USA) following the manufacturer’s instructions. Briefly, 100 ng of total RNA was fragmented using a thermal cycler. First-strand cDNA synthesis was carried out using the SuperScript II reverse transcriptase (Invitrogen, Carlsbad, CA, USA), followed by second-strand synthesis to generate double-stranded cDNA, which was then purified using Agencourt AMPure XP beads (Beckman Coulter Inc., Brea, CA, USA). Adapter indexes (TruSeq® RNA Single Indexes Set A, Illumina) were ligated to the cDNA fragments. After a purification step, Polymerase Chain Reaction (PCR) amplification was performed using primers provided in the TruSeq® RNA Library Prep for Enrichment kit.

Target regions were selected and enriched using the TruSeq® RNA Enrichment Kit (Illumina) and the Illumina Exome Panel—Enrichment Oligos Only (Illumina). Enrichment involved an initial hybridization reaction followed by purification using streptavidin-conjugated magnetic beads (Illumina). A second hybridization and purification step were then performed, and the final cDNA pool was amplified.

Library quality was assessed using the Tapestation 4,150 instrument with a D1000 ScreenTape assay (Agilent, Santa Clara, CA, USA). Finally, 1.42 pM of the cDNA library was sequenced using the Illumina NextSeq 550Dx instrument (Illumina) with the NextSeq 500/550 Mid Output Reagent Kit v2 (150 cycles) in paired-end mode.

### 2.6 Bioinformatics analysis

In this study, we evaluated the quality of the sequencing reads using FastQC v.0.12.0 (Babraham Institute, Cambridge, UK) (FastQC. A Quality Control Tool for High Throughput Sequence Data. Available online: https://qubeshub.org/resources/fastqc, accessed on 15 November 2024). To improve the quality of the reads, we then processed them with Trimmomatic v.0.40-rc1 (Usadel Lab, Aachen, Germany) (Bolger et al., 2014), which allowed us to remove adapter sequences and eliminate low-quality reads. Following this, the filtered reads were aligned to the hg38 v39 reference genome from GENCODE using the STAR RNA sequencing (RNA-seq) aligner 2.7.10a_alpha_220207 (New York, NY, USA) (Dobin et al., 2013). The most relevant scores of the analysis of each sample are reported in Supplementary Table S1. We calculated the transcript counts per gene using HTSeq v.0.13.5 (Anders et al., 2015). Differential gene expression analysis was conducted using the DESeq2 package v.1.36.0 (Love et al., 2014) in R v.4.2.0 (R Core Team). To minimize the risk of false positives, *p*-values were adjusted using the Benjamini–Hochberg procedure, with a q-value threshold of 0.05. Principal Component Analysis (PCA) plots in Supplementary Figures S1–S3 illustrate the clustering of samples within each experimental group. The resulting list of differentially expressed genes (DEGs) was further examined for overrepresented gene ontology terms using Panther (Available online: https://pantherdb.org/, accessed on 22 November 2024) (Mi et al., 2019), with unclassified terms excluded. Finally, pathway analysis of the DEGs was performed using the Kyoto Encyclopedia of Genes and Genomes (KEGG) database (Kanehisa and Goto, 2000).

### 2.7 Phage propagation

Engineered phages were obtained from M13 phage libraries constructed using the pC89 vector by cloning a random DNA insert between the third and fifth codon of the mature pVIII-encoding segments of gene VIII (Felici et al., 1991). Specifically, the 12CIII1 phage clone was selected from a 12-mer phage library and displays the 12-amino-acid sequence GGGCIEGPCLE, which is fused to the major coat protein (pVIII) and has been shown to detect IgG levels correlated with AD (De Plano et al., 2020).

The 12CIII1-engineered phage clone (Amp^+^) was propagated in the bacterial host *Escherichia coli* (New England Biolabs, Ipswich, Massachusetts) strain TG1 (Kan^−^, Amp^−^, lacZ^−^), as reported in our previous work (De Plano et al., 2020). Briefly, *E. coli* cultures were infected with the engineered phage and incubated in Luria-Bertani (LB) medium containing ampicillin (50 μg/mL) at 250 rpm on a rotary shaker at 37 °C until an Optical Density at 600 nm (OD600) of 0.2 was reached. Then, isopropylthio-*β*-galactoside (IPTG, 40 μg/mL) and helper phage M13K07 (Kan^+^, 10^9^ TU/mL) were added. The culture was incubated at 37 °C under static conditions for 15 min, followed by shaking at 250 rpm for 20 min.

After incubation, the culture was centrifuged at 8000 × g for 20 min, and the supernatant was removed and replaced with LB medium containing ampicillin (50 μg/mL) and kanamycin (10 μg/mL). The culture was then incubated overnight at 37 °C with shaking. The next day, the culture was centrifuged at 8000 × g for 20 min at 25 °C, and the supernatant was recovered and mixed with 25% (v/v) PEG-8000/NaCl solution. The mixture was cooled on ice for 4 h and precipitated by centrifugation at 15,000 × g for 45 min at 4 °C. The resulting pellet was resuspended in 10% (v/v) Tris-buffered saline (TBS; 7.88 g/L Tris hydrochloride and 8.77 g/L NaCl in deionized water), mixed again with 25% (v/v) PEG/NaCl, cooled on ice for 4 h, and centrifuged as described above.

The final pellet, containing phage particles, was resuspended in 10% (v/v) TBS, filtered through a 0.22 μm pore-size membrane (Millipore, Burlington, MA, USA), and heat-treated at 70 °C for 15 min before being stored at 4 °C.

### 2.8 Phage titration (transducing units per milliliter, TU/mL)

After phage propagation, tenfold serial dilutions of the phage suspension were prepared. Then, 10 μL from each dilution was added to sterile microcentrifuge tubes containing 90 μL of *E. coli* TG1 culture at an OD600 of 0.7. The tubes were incubated at 37 °C under static conditions for 15 min, followed by shaking at 250 rpm on a rotary shaker for 20 min. After incubation, 100 μL of each *E. coli* TG1/phage suspension was plated on LA plates containing ampicillin (50 μg/mL) and incubated overnight at 37 °C. Plates with 30–300 colonies were counted to determine the number of active viral particles capable of infecting the host cell and expressing the transgene. The number of active phage particles was expressed as TU/mL using the following equation:


TU/mL=(number of colonies)/volume(0.1mL)×dilution factor

For each assay, we used at least three biological replicates.

## 3 Results

### 3.1 12CIII binds to low molecular weight Aβ oligomers

We previously identified an engineered phage, 12CIII1, that recognizes conformation-specific Aβ autoantibodies in AD serum due to the GGGCIEGPCLE peptide displayed on the pVIII protein of the phage (Rizzo et al., 2023). To determine whether 12CIII1 can directly bind different assembly states of Aβ, we used two bioinformatic approaches: ZDOCK and iCn3D, which predict docking complexes and map the amino acids involved in the interaction, respectively (Pierce et al., 2014; Wang et al., 2020).

We first obtained known 3D Aβ structures from the RCSB PDB (PDB ID: 2NAO) (Walti et al., 2016) and, using YASARA software, we modeled the structure of low-molecular-weight Aβ oligomers. In the ZDOCK server, we used the Aβ models as ligands and the 3D model of the exposed region of the 12CIII1-pVIII protein as the receptor. ZDOCK employs an Fast Fourier Transform (FFT) algorithm to detect up to 2000 docking models, ranking them based on ZDOCK score values, which indicate binding capability and are derived from the interaction energy (Pierce et al., 2014; Malhotra et al., 2015; Aramyan et al., 2022). Based on these scores, we analyzed the top three predicted 3D docking models (Supplementary Figures S4–S6).

**Figure 1:**
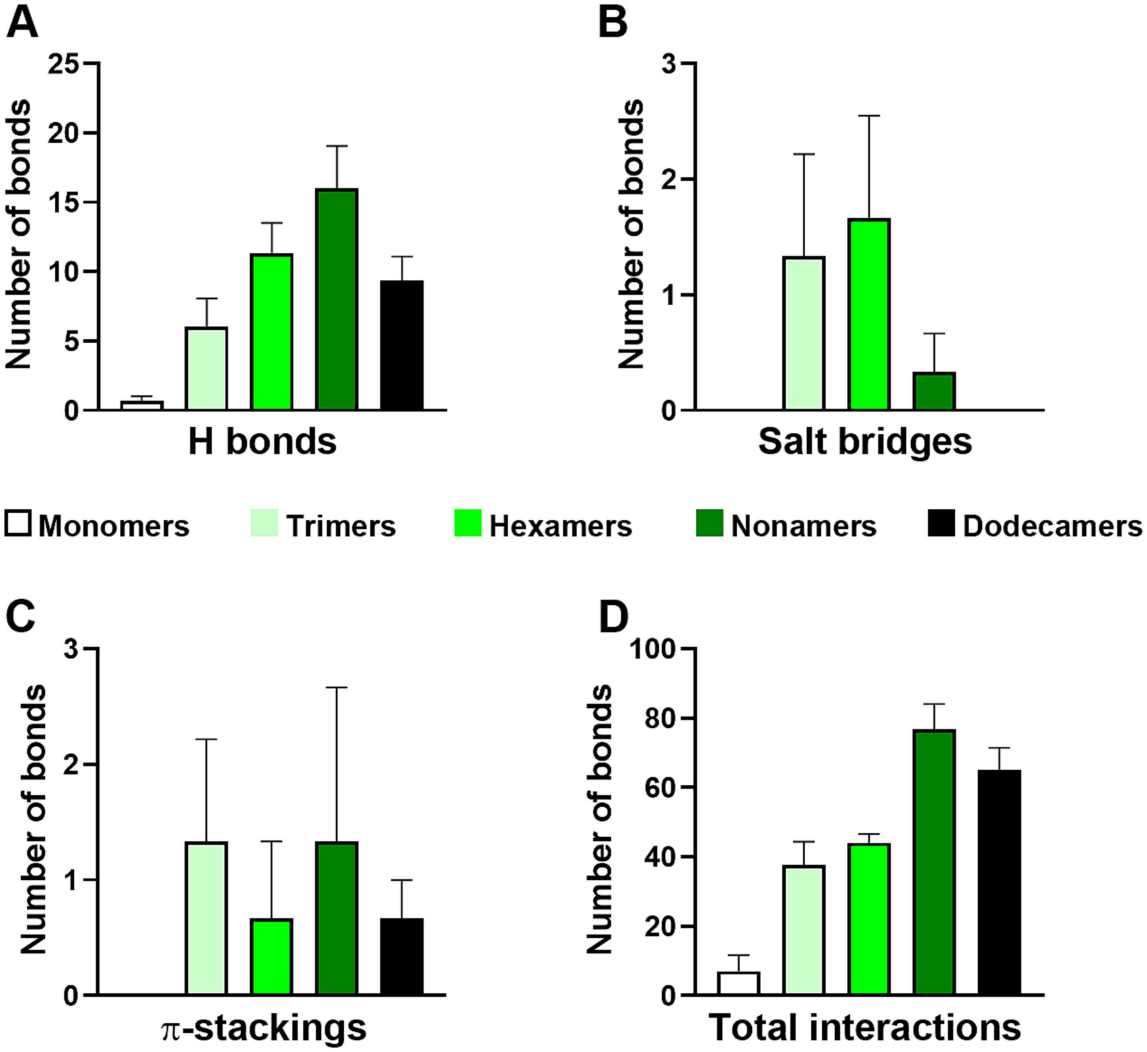
Frequency distribution of interactions. **(A-D)** The graph displays the frequency distribution of the most common non-covalent interactions, hydrogen bonds, salt bridges, and *π*-stacking, observed in the three docking sets between exposed region of 12CIII1 against different conformational states of amyloid-beta (Aβ) structures.

We quantified the average number and types of bonds formed between different Aβ assembly states and the engineered pVIII protein using the iCn3D server (Figure 1). Our analysis revealed that, except for Aβ monomers, all other Aβ assembly states formed a large number of Hydrogen-bond interactions (Figure 1A), which were the most common interactions across all three models. Specifically, the number of interactions gradually increased from monomers to nonamers, but then decreased in dodecamers (Figure 1A). Although hydrogen bonds have relatively low bond energy, they provide directionality to molecular interactions, which is critical for protein folding and molecular recognition, thereby contributing to the initial stability of the complexes. This section may be divided by subheadings. It should provide a concise and precise description of the experimental results, their interpretation, as well as the experimental conclusions that can be drawn.

Additionally, we found that all tested Aβ oligomers, but not Aβ monomers, exhibited salt-bridge and *π*-stacking interactions (Figures 1B,C). The presence of salt bridges, in combination with hydrogen bonds, enhances the physicochemical properties and protein folding of the molecular complex (Hubbard and Haider, 2010; Spassov et al., 2022). Overall, the total number of interactions was lowest for Aβ monomers, similar between trimers and hexamers, and highest in nonamers and dodecamers (Figure 1D). ZDOCK score comparisons confirmed that the strongest predicted interaction of the engineered pVIII protein occurred with Aβ nonamers, followed by dodecamers (Table 1).

**Table 1:** ZDOCK score values.

	Monomers	Trimers	Hexamers	Nonamers	Dodecamers
mod 1	1250.786	1280.252	1212.875	2129.467	2095.604
mod 2	1247.195	1213.152	1194.238	1985.576	2034.756
mod 3	1188.192	1190.282	1184.134	1972.939	2007.913

The table shows the score values of the first three docking models obtained between different conformation states of amyloid-beta (Aβ) structures and the exposed region of 12CIII1-pVIII-protein. Higher score values indicate better interaction between molecules based on poses and the energy values.

**Figure 2:**
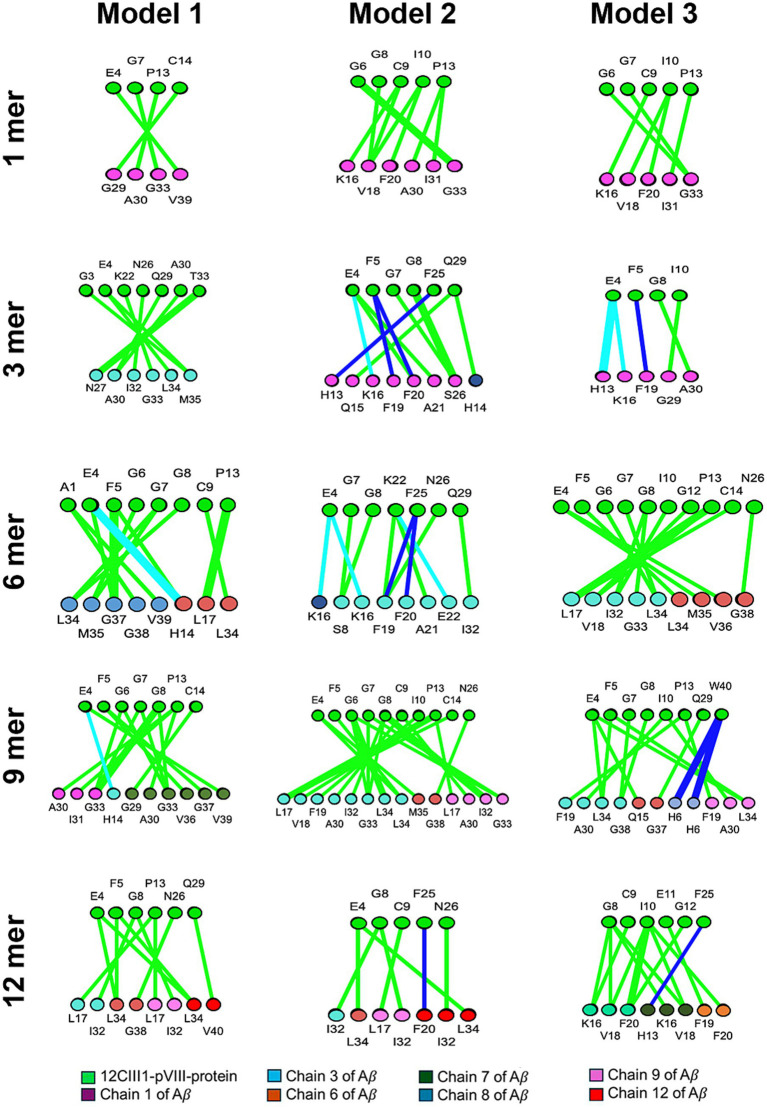
Amino acids involved in the interaction between amyloid-beta (Aβ) and the exposed region of 12CIII1. The image illustrates the amino acids participating in the interaction between different assembly states (1, 3, 6, 9, and 12) of Aβ and 12CIII1-pVIII-protein, as predicted using the iCn3D server. Each interaction is visualized as a ring, with amino acids from 12CIII1-pVIII-protein shown in green and those from Aβ in various colors.

We next sought to determine the precise locations of the interactions. Our analysis revealed that, in the exposed regions of the engineered pVIII protein, the N-terminal contains the amino acids primarily involved in the interaction. Specifically, Glycine 6, Glycine 7, Glycine 8, and Proline 13, followed by Cysteine 9 and Isoleucine 10, are the 12CIII1 peptide residues most involved in binding Aβ (Figure 2). Additionally, Glutamic Acid 4 and Phenylalanine 5, belonging to the N-terminal of the wild-type pVIII sequence, appear to contribute to the interaction (Figure 2).

In the Aβ sequence, the central region and C-terminal domain exhibited a higher number of amino acids involved in docking (Figure 2). Notably, within Aβ nonamers and dodecamers, we identified significantly enriched motifs predominantly localized in these regions (Figure 2). This is particularly relevant, as the central region of Aβ (amino acids 16–22) constitutes the hydrophobic core, which is crucial for self-assembly (Stankovic et al., 2020). Moreover, the hydrophobic C-terminal region of Aβ42 has been shown to play a key role in maintaining Aβ structural stability (Hughes et al., 2000). Further studies are needed to determine the specificity of the 12CIII1–Aβ interaction and to assess whether 12CIII1 also binds to other proteins with similar hydrophobic features.

The predicted binding free energies (ΔG) of the docked complexes, estimated using the PRODIGY web server (Xue et al., 2016), are summarized in Table 2. Overall, ΔG values ranged from −3.8 to −14.0 kcal·mol^−1^ across the different Aβ assembly states and engineered pVIII protein models, indicating a spectrum of binding affinities. Aβ monomers and small oligomers (1–3 mers) exhibited relatively weak interactions (ΔG ≈ −3.8 to −5.0 kcal·mol^−1^), whereas intermediate assemblies (6 mers) showed moderate binding. In contrast, larger Aβ aggregates displayed substantially stronger and more stable associations, with ΔG values reaching −10 kcal·mol^−1^ or lower, consistent with preferential binding of 12CIII1 to higher-order Aβ oligomeric species (Table 2).

**Table 2:** Predicted binding free energies (ΔG) of the docked complexes.

Binding free energy (ΔG, kcal/mol)	1 mer	3 mer	6 mer	9 mer	12 mer
Model 1	−4.8	−10.4	−8.9	−5.8	−11.1
Model 2	−4.9	−8.4	−9.6	−10.1	−14
Model 3	−3.8	−4.6	−6.5	−10.9	−5.8

The table reports ΔG values (kcal·mol^−1^) for the top three docking models obtained between different conformational states of Aβ and the exposed region of the 12CIII1-pVIII protein. More negative ΔG values indicate stronger and more stable binding. ΔG value ranges are classified as follows: −4 to −5 kcal·mol^−1^ (weak), −6 to −8 kcal·mol^−1^ (moderate), and < −9 kcal·mol^−1^ (high-affinity interactions).

### 3.2 12CIII1 protects against Aβ-induced toxicity

**Figure 3:**
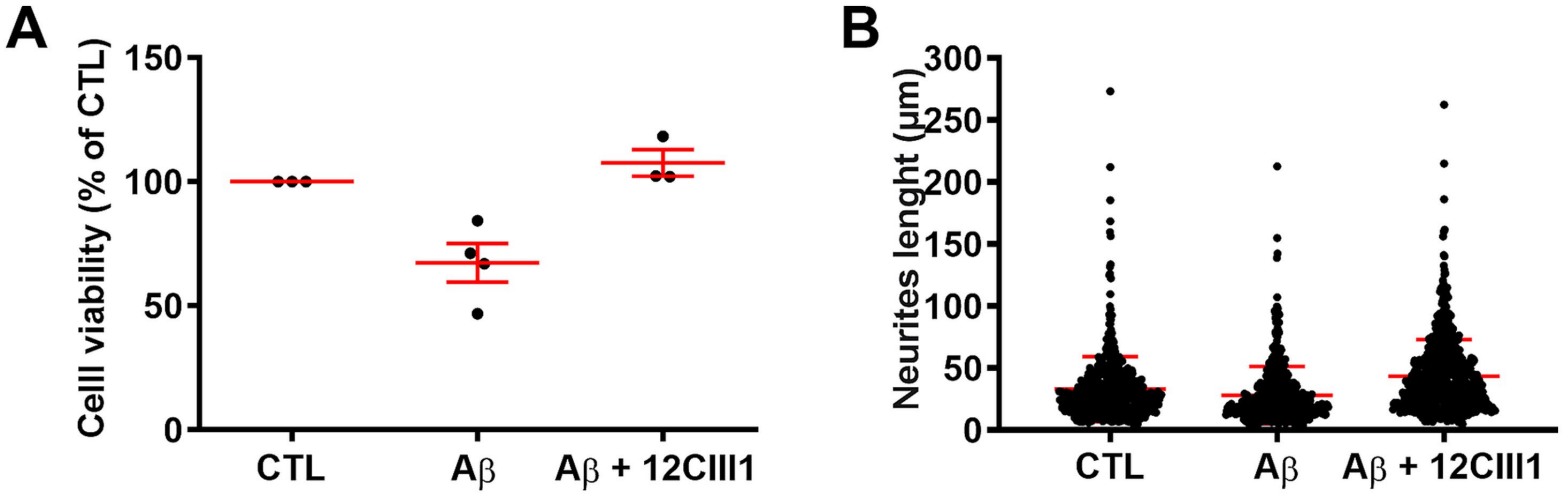
12CIII1 protects against amyloid-beta (Aβ)-induced toxicity. **(A)** MTT assay assessing the viability of SH-SY5Y cells treated with Aβ alone or Aβ plus 12CIII1. Aβ treatment significantly reduced cell viability compared to Control (CTL) (*p* < 0.001). Co-treatment with 12CIII1 restored viability (*p* < 0.001 compared to the Aβ-treated group). **(B)** Quantification of neurite length using ImageJ. Aβ treatment significantly reduced neurite length compared to untreated control cells (*p* < 0.001). Co-treatment with 12CIII1 rescued neurite outgrowth (*p* < 0.001 compared to the Aβ-treated group). Data were analyzed by one-way Analysis of Variance (ANOVA) followed by *post hoc* testing.

Using a computational approach, we demonstrated that 12CIII1 binds to different assembly states of Aβ, showing minimal affinity for Aβ monomers but stronger interactions with nonamers and dodecamers. This finding prompted us to investigate whether 12CIII1 could protect SH-SY5Y cells from Aβ-induced toxicity. To assess this, we evaluated cell viability and neurite length under different treatment conditions. We found that treatment with 2 μM Aβ42 significantly reduced cell viability (*p* < 0.0001; Figure 3A). However, preincubation with 12CIII1, added to cells 1 hour before Aβ42 exposure, prevented Aβ-induced toxicity (Figure 3A).

We next examined the effects of Aβ42 on neuronal morphology. Our results showed that Aβ42 led to a statistically significant reduction in neurite length compared to the control group (Figure 3B). Remarkably, 12CIII1 not only protected against Aβ-induced neurite shortening but also enhanced neurite outgrowth to levels exceeding those of the control (*p* < 0.0001). Together, these findings highlight the potency of 12CIII1 in mitigating Aβ toxicity, protecting SH-SY5Y cells by enhancing cell viability and promoting neurite extension.

### 3.3 Transcriptomic analysis of 12CIII1-mediated protection against Aβ toxicity

**Figure 4:**
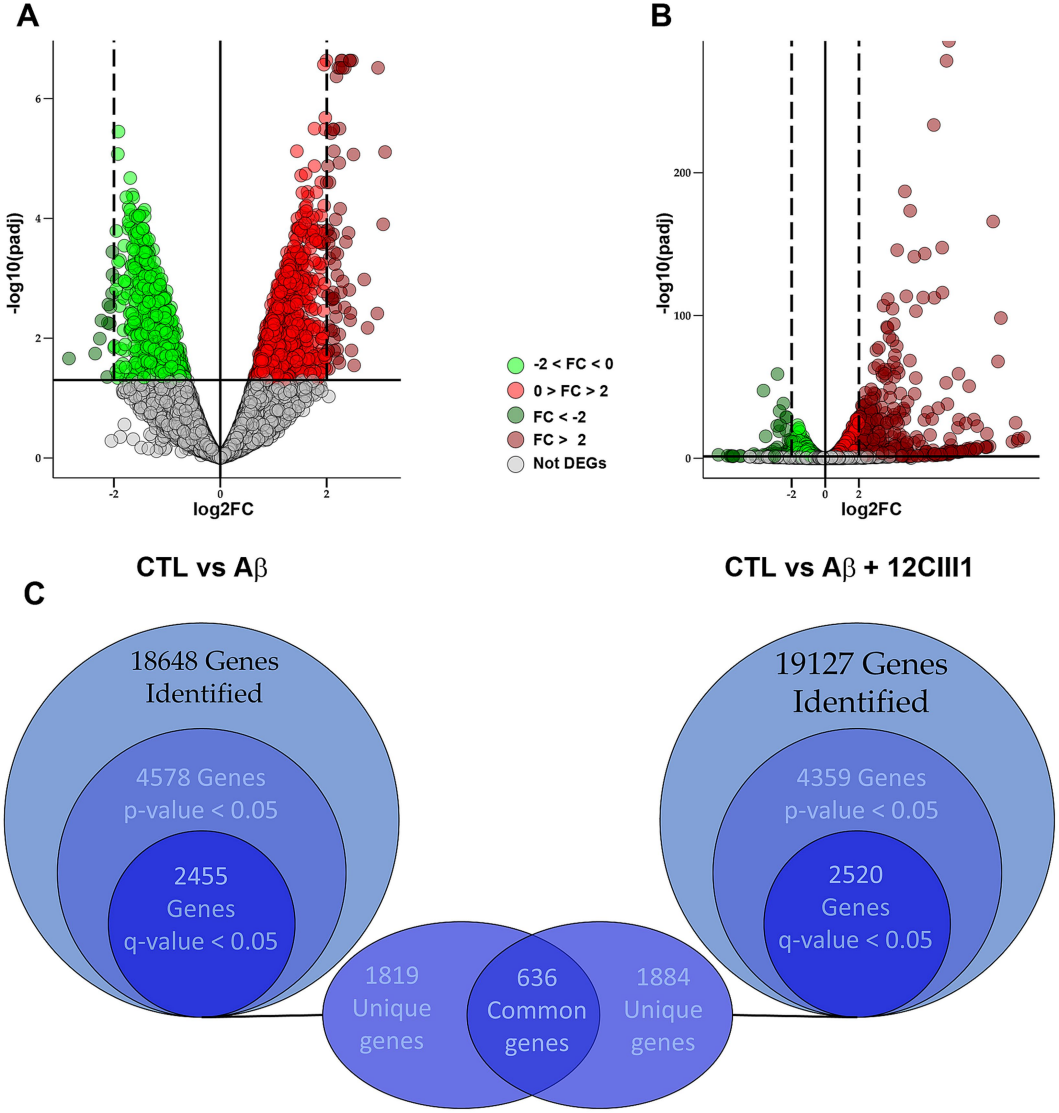
Differential gene expression analysis between amyloid-beta (Aβ) and Aβ + 12CIII1555 treatment. Volcano plots comparing transcriptome profiles: **(A)** control vs. Aβ-treated cells and **(B)** control vs. Aβ + 12CIII1-treated cells. Each dot represents an analyzed gene, with colors indicating fold-change patterns: light or dark green for downregulated genes (from 0 to −2 or lower than −2, respectivelly) and light or dark red for upregulated genes (from 0 to 2 or higher than 2, respectivelly). When the corrected *p*-value is higher than 0.5, the genes is defined as non-differentially expressed and it is shown in grey. **(C)** Euler diagram illustrating the number of differentially expressed genes in both comparisons, highlighting the shared deregulated genes.

To better understand how 12CIII1 protects against Aβ-induced toxicity, we performed RNA-seq experiments using total RNA isolated from SH-SY5Y cells treated with Aβ or Aβ + 12CIII1, as detailed in the Methods section. Our initial goal was to identify genes altered by Aβ treatment in SH-SY5Y cells. A total of 18,648 transcripts were identified, and the expression levels of 4,578 genes were significantly different between the two groups (*p* < 0.05). After post-hoc correction to control the false discovery rate, 2,455 genes retained an adjusted *p*-value (*q* < 0.05; Figures 4A,C).

To assess the impact of 12CIII1 on the transcriptomic profile, we performed a similar analysis, comparing the transcriptome of control cells to that of cells exposed to Aβ and 12CIII1. We identified 19,127 transcripts, with 4,359 genes showing significant differential expression between these two groups (*p* < 0.05). Of these, 2,520 genes maintained a q-value < 0.05 after post-hoc correction (Figures 4B,C).

We hypothesized that some of the 2,455 DEGs between SH-SY5Y control (CTL) and SH-SY5Y-Aβ-treated cells might be involved in Aβ-induced toxicity. To explore this, we examined which of these 2,455 genes were not differentially expressed when comparing SH-SY5Y-CTL to SH-SY5Y-Aβ + 12CIII1, given that 12CIII1 mitigates Aβ toxicity. We identified 1,819 genes whose expression levels were altered following Aβ exposure but remained unchanged in the presence of Aβ + 12CIII1 (Figure 4C and Supplementary file 1 for the full list). Among these, the most strongly upregulated and downregulated genes by Aβ, whose expression was restored to baseline by 12CIII1, are listed in Table 3.

**Table 3:** Top 20 genes with the highest and lowest fold changes upon Aβ exposure whose expression reverted to baseline after Aβ + 12CIII1 treatment.

Gene	Fold change	*q*-value
TNNT3	3.06	1.26 × 10^−4^
KRT18P6	2.95	3.87 × 10^−3^
KRT17P8	2.77	6.74 × 10^−3^
MFAP1P1	2.71	1.05 × 10^−3^
YBX1P4	2.52	2.85 × 10^−2^
MTND2P28	2.50	8.59 × 10^−6^
RPL23AP35	2.47	1.60 × 10^−2^
RPL23AP43	2.45	3.80 × 10^−3^
DSPP	2.44	5.01 × 10^−3^
5_8S_rRNA	2.43	1.57 × 10^−3^
ENSG00000280287	2.41	1.75 × 10^−4^
GOLGA6A	2.36	2.48 × 10^−4^
RNA5-8SN4	2.34	3.07 × 10^−3^
AK2P2	2.24	8.66 × 10^−3^
ENSG00000261635	2.24	1.19 × 10^−5^
FIGLA	2.24	2.58 × 10^−2^
ENSG00000233230	2.20	2.17 × 10^−2^
FOXD4L3	2.20	8.91 × 10^−4^
PGAM1P10	2.20	1.55 × 10^−2^
SMARCE1P5	2.20	3.92 × 10^−4^
ENSG00000218175	−2.35	1.81 × 10^−2^
IGHV3-36	−2.27	1.02 × 10^−2^
TSLP	−2.24	5.07 × 10^−3^
C4orf46	−2.13	4.45 × 10^−2^
TRIQK	−2.10	2.74 × 10^−3^
PHF5A	−2.07	3.02 × 10^−3^
PUS3	−2.03	8.75 × 10^−4^
MAD2L1	−1.99	1.44 × 10^−2^
OXR1	−1.99	1.36 × 10^−2^
GGCT	−1.96	3.57 × 10^−2^
MPC2	−1.95	1.62 × 10^−4^
CISD1	−1.93	4.61 × 10^−2^
NDUFB6	−1.92	5.34 × 10^−4^
PIGA	−1.92	4.02 × 10^−2^
MRPL15	−1.89	2.90 × 10^−3^
NDUFAB1	−1.89	4.97 × 10^−4^
RPL3P4	−1.87	1.50 × 10^−2^
DMAC1	−1.85	2.05 × 10^−3^
DDX28	−1.82	7.37 × 10^−3^
NUDCD2	−1.81	2.18 × 10^−3^

Additionally, among the 2,520 DEGs in the CTRL vs. Aβ + 12CIII1 comparison, we identified 1,884 genes whose expression levels were altered in cells treated with Aβ + 12CIII1 but not in those exposed to Aβ alone (Figure 4C and Supplementary file 1 for the full list). We hypothesize that some of the 1,819 genes that were differentially expressed following Aβ exposure but returned to baseline in the Aβ + 12CIII1 group may contribute to the protective effects of 12CIII1 against Aβ toxicity.

### 3.4 Functional classification of genes involved in Aβ-induced toxicity

To gain insight into the biological functions of the 1,819 genes whose expression was altered by Aβ exposure but restored to control levels in the presence of Aβ + 12CIII1, we performed Gene Ontology (GO) over-representation analysis using the PANTHER classification system (v19.0). PANTHER categorizes genes and proteins based on molecular function, biological process, cellular component, and protein class, enabling high-throughput analysis of large datasets.

**Figure 5:**
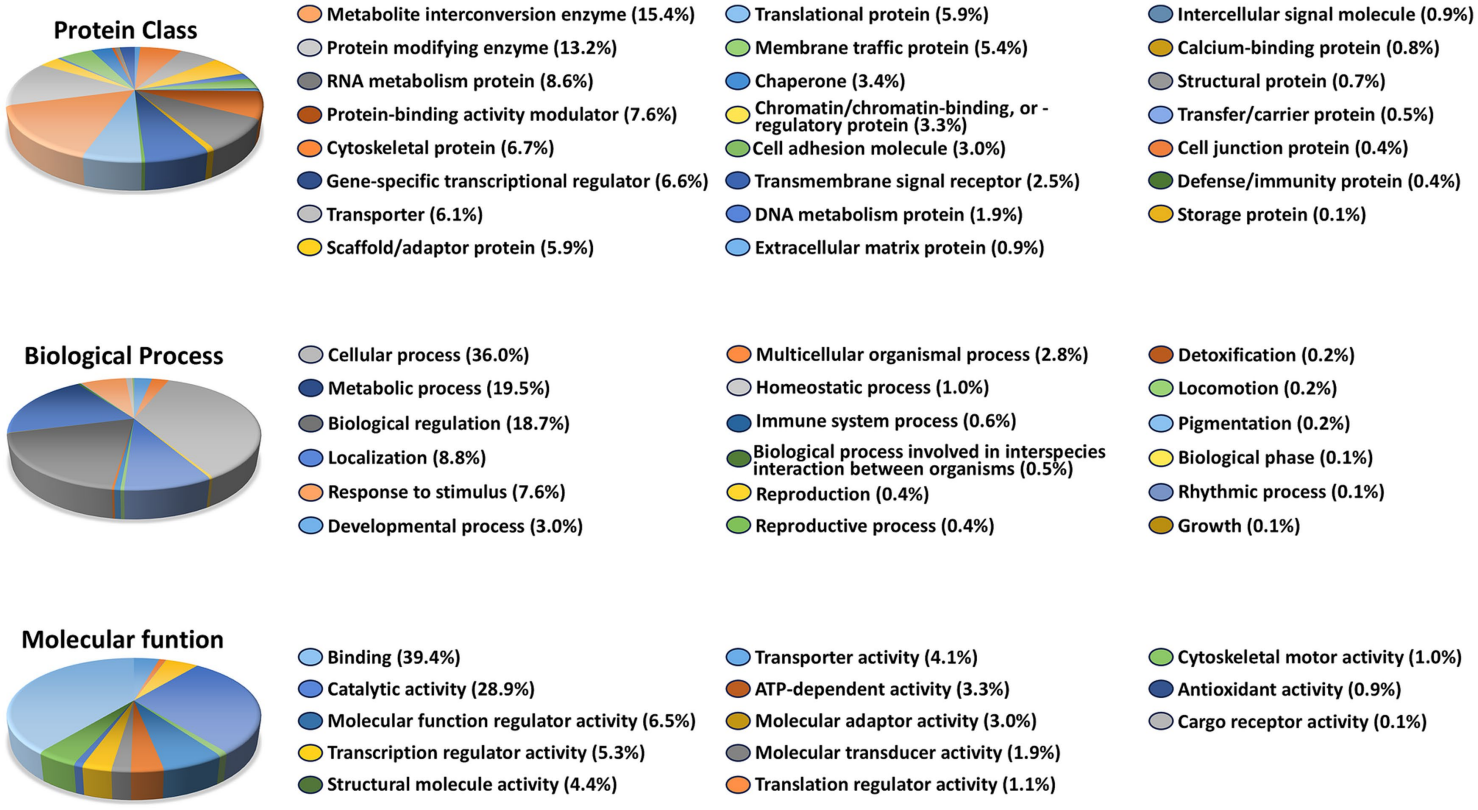
Functional pathways affected by amyloid-beta (Aβ) Exposure: Gene Ontology (GO Analysis). GO over-representation analysis based on 1,819 unique genes that exhibited altered expression levels following Aβ exposure but not in the Aβ + 12CIII1-treated condition.

GO analysis revealed that these 1,819 genes belong to diverse protein classes (Figure 5). The largest proportion of genes falls into the categories of metabolite interconversion enzymes and protein-modifying enzymes. This suggests that genes involved in energy production, biosynthesis, detoxification, and post-translational modifications are disproportionately affected by Aβ exposure but are restored to normal expression levels by 12CIII1. This notion is further supported by the biological process classification, which indicates that the highest percentage of genes are associated with cellular and metabolic processes.

When classifying the 1,819 genes based on the molecular function of their encoded proteins, we found that most are involved in metabolism, transport, growth regulation, and gene expression regulation, predominantly exhibiting catalytic or binding activity (Figure 5). Together, these results indicate that genes significantly affected by A*β* exposure play key roles in regulating metabolic pathways and post-translational modifications. The dysregulation of these gene classes may underlie Aβ-induced toxicity.

**Figure 6:**
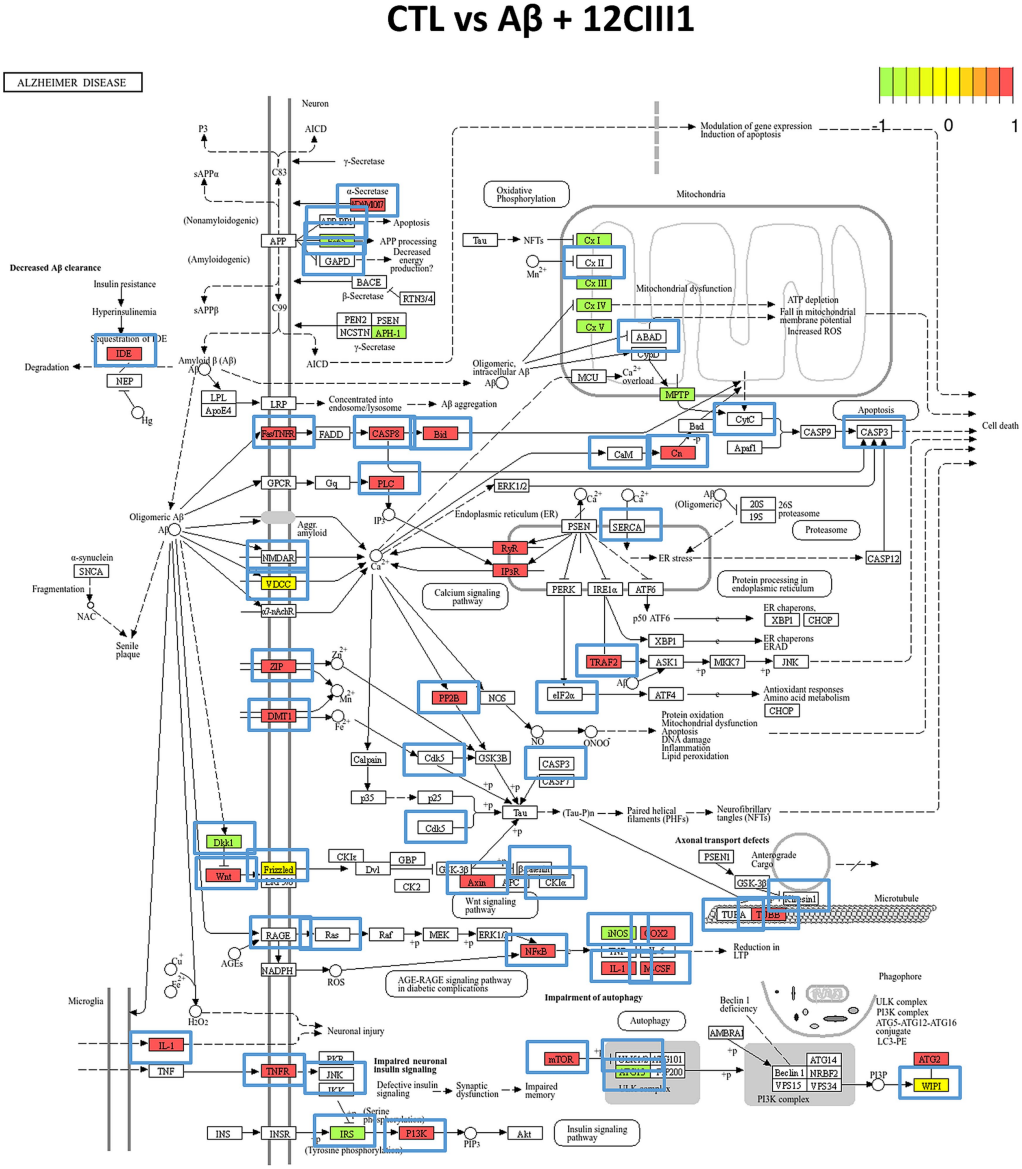
Alzheimer’s disease (AD) pathway analysis. The pathway (KEGG ID: hsa05010) was enriched with Differentially Expressed Genes (DEGs) identified in our transcriptomic analysis. Green boxes represent proteins associated with DEGs downregulated in the CTL vs. Aβ + 12CIII1 comparison, while red boxes indicate upregulated DEGs. Yellow boxes highlight DEGs that show both up- and downregulation. Blue boxes outside the proteins indicate proteins whose DEG regulation pattern changes in the CTL vs. Aβ comparison.

We further examined the cellular localization and pathway involvement of these genes using the KEGG pathway database. In particular, Figure 6 depicts the DEGs mapped to the AD pathway (KEGG ID: hsa05010). We highlighted DEGs that were differentially expressed in the CTL vs. Aβ + 12CIII1 comparison, as well as those whose expression patterns changed in the CTL vs. Aβ comparison.

## 4 Discussion

In this study, we demonstrate that the engineered phage 12CIII1 binds directly to Aβ, mitigates Aβ-induced toxicity in SH-SY5Y cells, and restores Aβ-driven alterations in gene expression. These findings provide novel insights into the therapeutic potential of phage-displayed peptides for AD and suggest that 12CIII1 may modulate cellular responses to Aβ toxicity through specific molecular interactions.

Our bioinformatic analyses revealed that 12CIII1 exhibits preferential binding to oligomeric forms of Aβ rather than monomers, with the strongest interaction observed for Aβ nonamers and dodecamers. This is a critical finding, as Aβ oligomers are widely recognized as the most neurotoxic species in AD pathology (Li and Selkoe, 2020; Min Kaung Min Kaung Wint et al., 2024; Zabala-Rodriguez et al., 2025). The ability of 12CIII1 to target these toxic conformations may underlie its protective effects on neuronal cells. Further work is required to dissect the molecular pathways through which 12CIII1 rescues gene expression profiles. A plausible mechanism is that 12CIII1 binds to Aβ oligomers outside the cells thus preventing their interaction with neuronal receptors and the subsequent activation of signaling networks that drive transcriptional dysregulation. To this end, we identified key residues within the 12CIII1 peptide that mediate Aβ binding, providing a foundation for future studies aimed at optimizing peptide affinity and specificity. Additionally, our data highlight that interactions occur predominantly within the hydrophobic core and C-terminal domain of Aβ, regions essential for aggregation and toxicity (Lu et al., 2019; Tung et al., 2019; Itoh et al., 2022). This observation suggests that 12CIII1 may interfere with Aβ self-assembly, thereby reducing its pathogenic potential. While our analyses focused on Aβ species, future studies will be needed to determine whether 12CIII1 discriminates Aβ oligomers from other proteins with β-sheet–rich hydrophobic cores. Given the structural similarities among oligomeric intermediates formed by different amyloidogenic proteins, it will be important to explore whether 12CIII1 also recognizes shared conformational motifs present in other neurotoxic aggregates. Such investigations will help clarify the degree of specificity and the potential broader applicability of 12CIII1 as a modulator of protein misfolding toxicity.

Functionally, we observed that 12CIII1 protects SH-SY5Y cells from Aβ-induced toxicity by preserving cell viability and promoting neurite outgrowth. This neuroprotective effect is consistent with previous studies demonstrating that targeting Aβ oligomers can prevent synaptic dysfunction and neuronal death (Goncalves et al., 2024; Min Kaung Wint et al., 2024; Wang et al., 2025; Zabala-Rodriguez et al., 2025). Notably, neurite outgrowth enhancement in the presence of 12CIII1 suggests that this phage may not only counteract Aβ toxicity but also promote neuronal resilience. While our functional assays demonstrate that 12CIII1 protects neuronal viability and preserves neurite length in the presence of Aβ, we acknowledge that additional markers of synaptic integrity and oxidative stress were not assessed in this study. Future work, both in primary neurons or *in vivo* models of AD, incorporating these endpoints will be important to fully characterize the cellular mechanisms underlying 12CIII1-mediated neuroprotection and to refine interpretation of its functional effects.

Our transcriptomic analysis provided additional mechanistic insights, revealing that 12CIII1 prevents a substantial subset of Aβ-driven gene expression alterations. A total of 1,819 genes that were dysregulated upon Aβ exposure were restored to control levels in the presence of 12CIII1. Functional classification of these genes indicates that they are predominantly involved in metabolism, cellular stress responses, and protein modification processes, all of which are known to be disrupted in AD (Trovato Salinaro et al., 2014; Uddin et al., 2021). In particular, genes encoding metabolite interconversion enzymes and protein-modifying enzymes were among the most affected, suggesting that Aβ toxicity may impair essential metabolic and regulatory pathways, which are subsequently restored by 12CIII1 treatment. This aligns with recent findings implicating metabolic dysfunction as a key driver of neurodegeneration in AD (Abdalla, 2024; Feng et al., 2025; Kaur et al., 2025). Of note, among the 20 most upregulated genes by Aβ, whose expression was restored to baseline by 12CIII1 (Table 3), five (25%) are directly involved in mitochondrial metabolism: NDUFB6, a subunit of NADH:ubiquinone oxidoreductase (Complex I); NDUFAB1, an accessory subunit of Complex I; MRPL15, a mitochondrial ribosomal protein; MPC2, a component of the mitochondrial pyruvate carrier complex; and CISD1, which regulates mitochondrial iron homeostasis and oxidative capacity. These data further strengthen the link between mitochondrial dysfunction and Aβ-induced toxicity. Given the increasing recognition that early metabolic and mitochondrial impairments precede overt amyloid and tau pathology, our findings suggest that 12CIII1 may act upstream by stabilizing cellular energy homeostasis. Thus, by mitigating Aβ-induced metabolic stress, 12CIII1 could contribute to preserving neuronal resilience in the early AD stages.

Interestingly, 12CIII1 not only reversed Aβ-induced gene expression changes but also led to differential expression of 1,844 genes that were not affected by Aβ alone. This suggests that 12CIII1 may actively modulate additional cellular pathways beyond those directly perturbed by Aβ. Whether these changes contribute to the enhanced neurite outgrowth observed in our study remains an open question. Further analysis, including pathway enrichment and network-based approaches, may elucidate the specific cellular programs influenced by 12CIII1.

A limitation of the present study is the absence of a non-binding or scrambled phage control. Nevertheless, the phage clone 12CIII1 was previously identified and validated by our group for its specific ability to discriminate AD from non-AD sera (Rizzo et al., 2023). Therefore, it is reasonable to assume that the effects observed here are sequence-specific and reflect genuine interactions between 12CIII1 and Aβ oligomers.

The present findings suggest that the 12CIII1 phage exerts protective effects against Aβ-induced neurotoxicity and prevents Aβ-driven alterations in gene expression, indicating a potential therapeutic role in modulating the downstream consequences of Aβ pathology. In this context, it is important to position the phage display strategy within the broader landscape of anti-Aβ interventions. Compared to monoclonal antibodies, which have shown variable clinical efficacy and are often limited by high production costs, restricted brain penetration, and immune-related adverse effects, phage-displayed peptides offer a versatile and cost-effective alternative. Phage particles can be engineered to display conformationally constrained peptides that mimic antibody-binding epitopes while maintaining high target specificity. Moreover, the multivalent display of peptides on the phage surface can enhance avidity toward aggregated Aβ species, potentially improving neutralization efficiency. When contrasted with D-peptides and nanobodies, phage-displayed peptides are easier to generate through combinatorial selection and can serve as scaffolds for subsequent conversion into smaller, more drug-like entities. Nevertheless, it must be acknowledged that the use of intact bacteriophages as therapeutic agents faces practical limitations, including immunogenicity, stability in systemic circulation, and challenges in blood–brain barrier penetration. Future work will therefore focus on isolating the active peptide sequence from 12CIII1 and optimizing it for delivery *in vivo*. Overall, these data highlight the promise of phage display–derived ligands as a complementary approach to existing anti-Aβ strategies, with the potential to yield novel therapeutic leads capable of targeting early molecular events in AD.

## 5 Conclusion

This study contributes to the growing body of research supporting the use of phage display technology to target Aβ aggregates associated with AD. By demonstrating that 12CIII1 interacts with toxic Aβ species and mitigates their neurotoxic effects, we provide new insights into potential therapeutic pathways and the development of personalized therapies for AD. In the future, integrating this technology into clinical applications could offer a novel strategy for treating Aβ-related disorders, potentially enabling more targeted and effective therapies. However, further refinement of these strategies is essential to ensure their safety and efficacy in more complex biological models.

## Data Availability

The sequencing data have been deposited on Sequence Read Archive: ID PRJNA136933.

## Footnotes

1
https://www.rcsb.org

2
https://zdock.wenglab.org

3
https://www.ncbi.nlm.nih.gov/Structure/icn3d/full.html

## Supplementary material

SUPPLEMENTARY FIGURE S1 Principal component analysis of CTL vs Aβ samples. The plot highlights the two components with the highest variance among the samples included in the comparison. The first principal component (PC1) clearly separates the two groups (CTL samples in red; Aβ samples in blue).

SUPPLEMENTARY FIGURE S2 Principal component analysis of CTL vs Aβ+12CIII1 samples. The plot highlights the two components with the highest variance among the samples included in the comparison. The first principal component (PC1) clearly separates the two groups (CTL samples in red; Aβ+12CIII1 samples in blue).

SUPPLEMENTARY FIGURE S3 Principal component analysis of Aβ vs Aβ+12CIII1 samples. The plot highlights the two components with the highest variance among the samples included in the comparison. The first principal component (PC1) clearly separates the two groups (Aβ samples in red; Aβ+12CIII1 samples in blue).

SUPPLEMENTARY FIGURE S4 Predictive interaction between Aβ and the exposed region of 12CIII1 (Model 1). The image depicts 3D docking models of different assembly states (1, 3, 6, 9, and 12) of Aβ (grey) interacting with the exposed region of 12CIII1 (yellow), obtained using the ZDOCK server. Each structure is displayed in cartoon style, with the amino acids involved in the interaction highlighted: residues from 12CIII1 are shown in green, while those from Aβ are represented in various colors using the Ball & Stick style.

SUPPLEMENTARY FIGURE S5 Predictive interaction between Aβ and the 12CIII1 foreign peptide (Models 2). These images depict 3D docking models of different assembly states (1, 3, 6, 9, and 12) of Aβ (grey) interacting with the exposed region of 12CIII1-pVIII-protein (yellow), obtained using the ZDOCK server. Each structure is displayed in cartoon style, with amino acids involved in the interaction highlighted: residues from 12CIII1-pVIII-protein are shown in green, while those from Aβ are in various colors using the Ball & Stick style.

SUPPLEMENTARY FIGURE S6 Predictive interaction between Aβ and the 12CIII1 foreign peptide (Models 3). These images depict 3D docking models of different assembly states (1, 3, 6, 9, and 12) of Aβ (grey) interacting with the exposed region of 12CIII1-pVIII-protein (yellow), obtained using the ZDOCK server. Each structure is displayed in cartoon style, with amino acids involved in the interaction highlighted: residues from 12CIII1-pVIII-protein are shown in green, while those from Aβ are in various colors using the Ball & Stick style.

## Glossary

AD: Alzheimer’s disease
Aβ: Amyloid-beta
PDB: Protein Data Bank
DOPE: Discrete Optimized Protein Energy
3D: Three-Dimensional
FFT: Fast Fourier Transform
CTL: Control
DEG(s): Differentially Expressed Gene(s)
GO: Gene Ontology
KEGG: Kyoto Encyclopedia of Genes and Genomes
SH-SY5Y: Human neuroblastoma cell line SH-SY5Y
DMEM/F-12: Dulbecco’s Modified Eagle Medium/Nutrient Mixture F-12
FBS: Fetal Bovine Serum
RA: Retinoic Acid
MTT: 3-(4,5-dimethylthiazol-2-yl)-2,5-diphenyltetrazolium bromide
DMSO: Dimethyl Sulfoxide
RNA-seq: RNA sequencing
cDNA: Complementary DNA
PCR: Polymerase Chain Reaction
TU/mL: Transducing Units per milliliter
LB: Luria-Bertani medium
IPTG: Isopropyl β-D-1-thiogalactopyranoside
TBS: Tris-buffered saline
OD600: Optical Density at 600 nm
ANOVA: Analysis of Variance
