# Supplementary figures and images for: A phage displaying an Aβ-interacting peptide mitigates neurotoxicity and prevents Aβ-driven gene expression changes
Source: Front Mol Neurosci. 2025 Dec 10;18:1716626. doi: 10.3389/fnmol.2025.1716626 (PMC12728043; doi:10.3389/fnmol.2025.1716626)

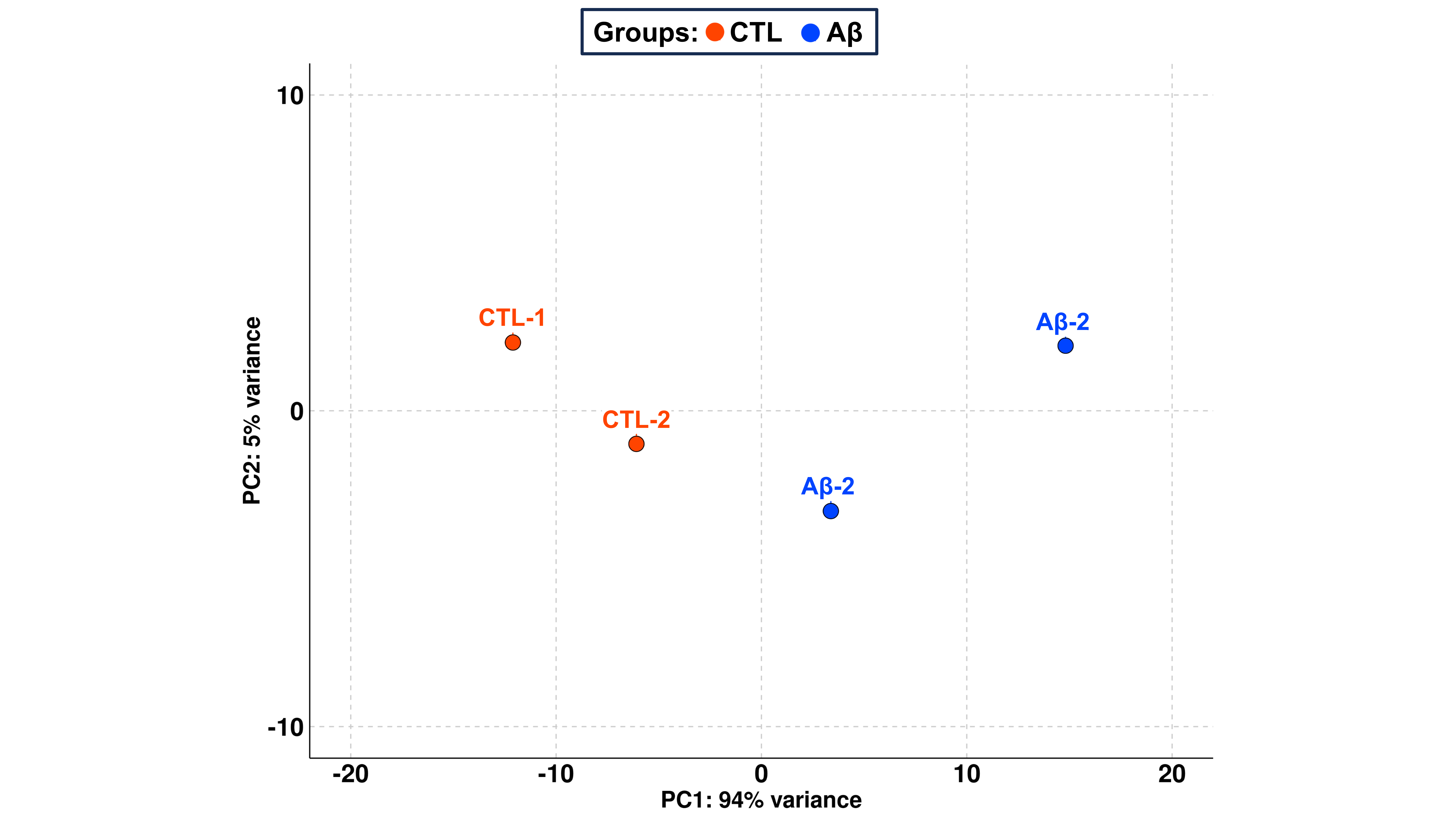

Supplement: SUPPLEMENTARY FIGURE S1 — Principal component analysis of CTL vs Aβ samples. The plot highlights the two components with the highest variance among the samples included in the comparison. The first principal component (PC1) clearly separates the two groups (CTL samples in red; Aβ samples in blue). [file Image_1.TIF]

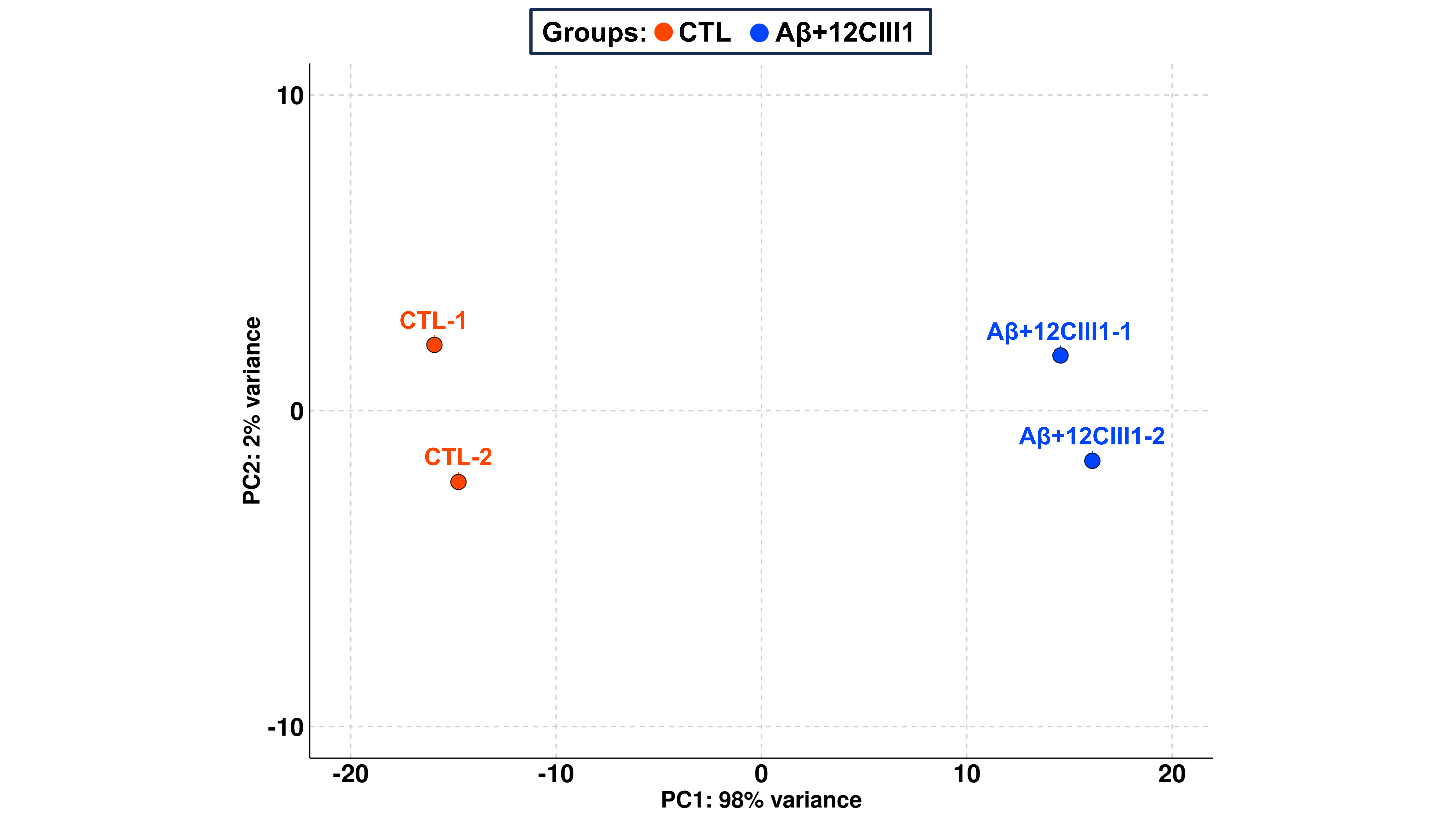

Supplement: SUPPLEMENTARY FIGURE S2 — Principal component analysis of CTL vs Aβ+12CIII1 samples. The plot highlights the two components with the highest variance among the samples included in the comparison. The first principal component (PC1) clearly separates the two groups (CTL samples in red; Aβ+12CIII1 samples in blue). [file Image_2.TIF]

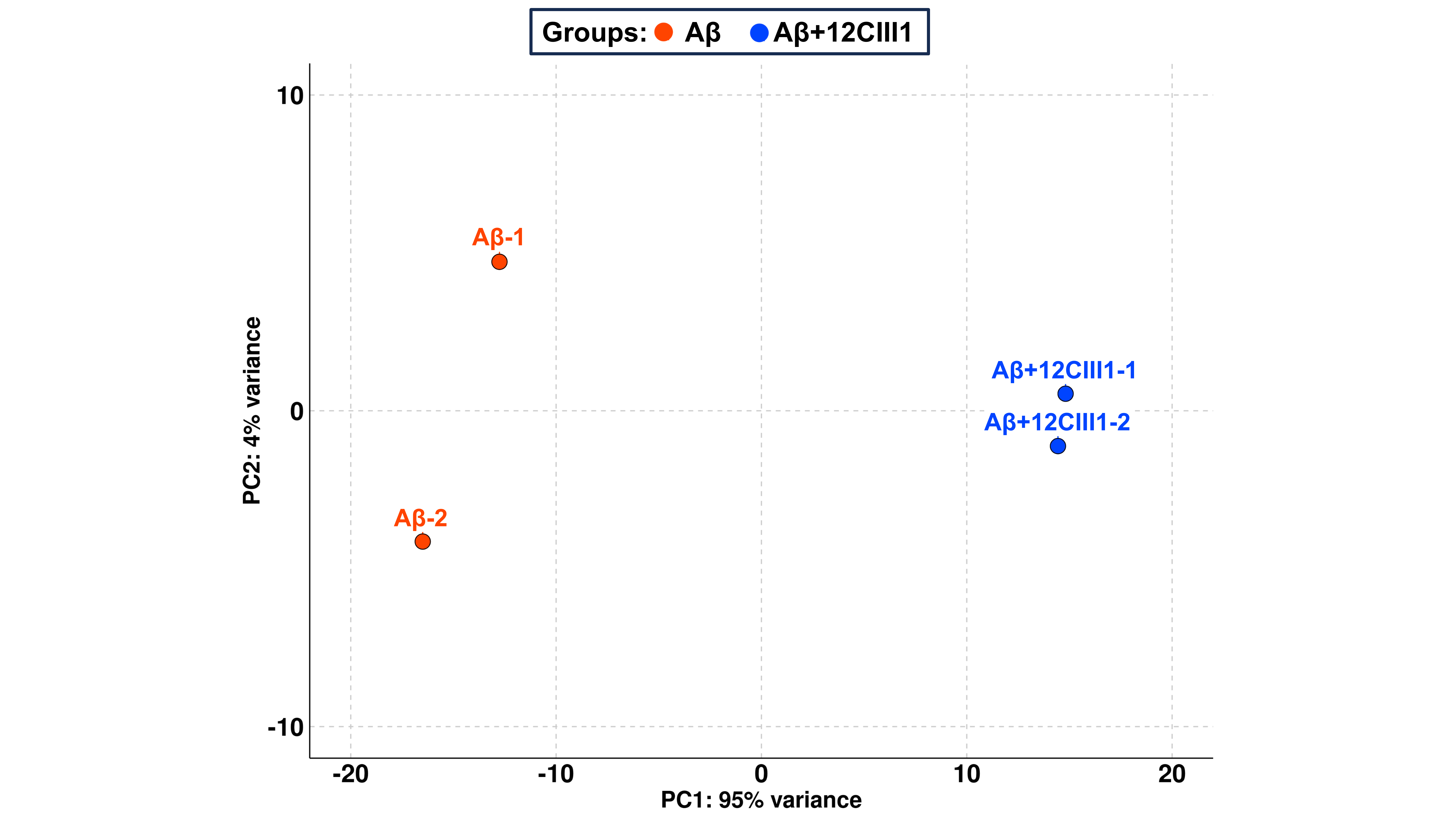

Supplement: SUPPLEMENTARY FIGURE S3 — Principal component analysis of Aβ vs Aβ+12CIII1 samples. The plot highlights the two components with the highest variance among the samples included in the comparison. The first principal component (PC1) clearly separates the two groups (Aβ samples in red; Aβ+12CIII1 samples in blue). [file Image_3.TIF]

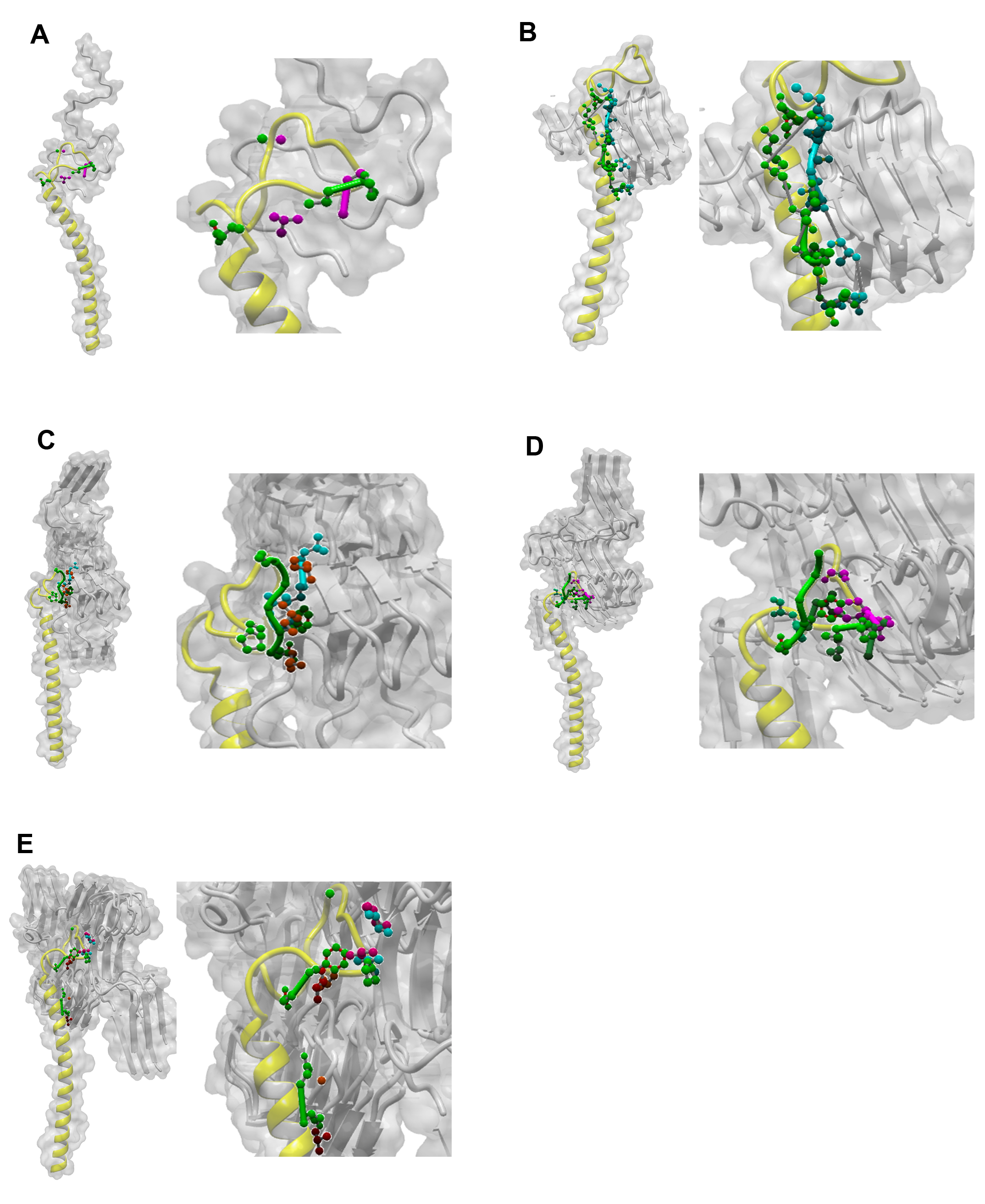

Supplement: SUPPLEMENTARY FIGURE S4 — Predictive interaction between Aβ and the exposed region of 12CIII1 (Model 1). The image depicts 3D docking models of different assembly states (1, 3, 6, 9, and 12) of Aβ (grey) interacting with the exposed region of 12CIII1 (yellow), obtained using the ZDOCK server. Each structure is displayed in cartoon style, with the amino acids involved in the interaction highlighted: residues from 12CIII1 are shown in green, while those from Aβ are represented in various colors using the Ball & Stick style. [file Image_4.TIF]

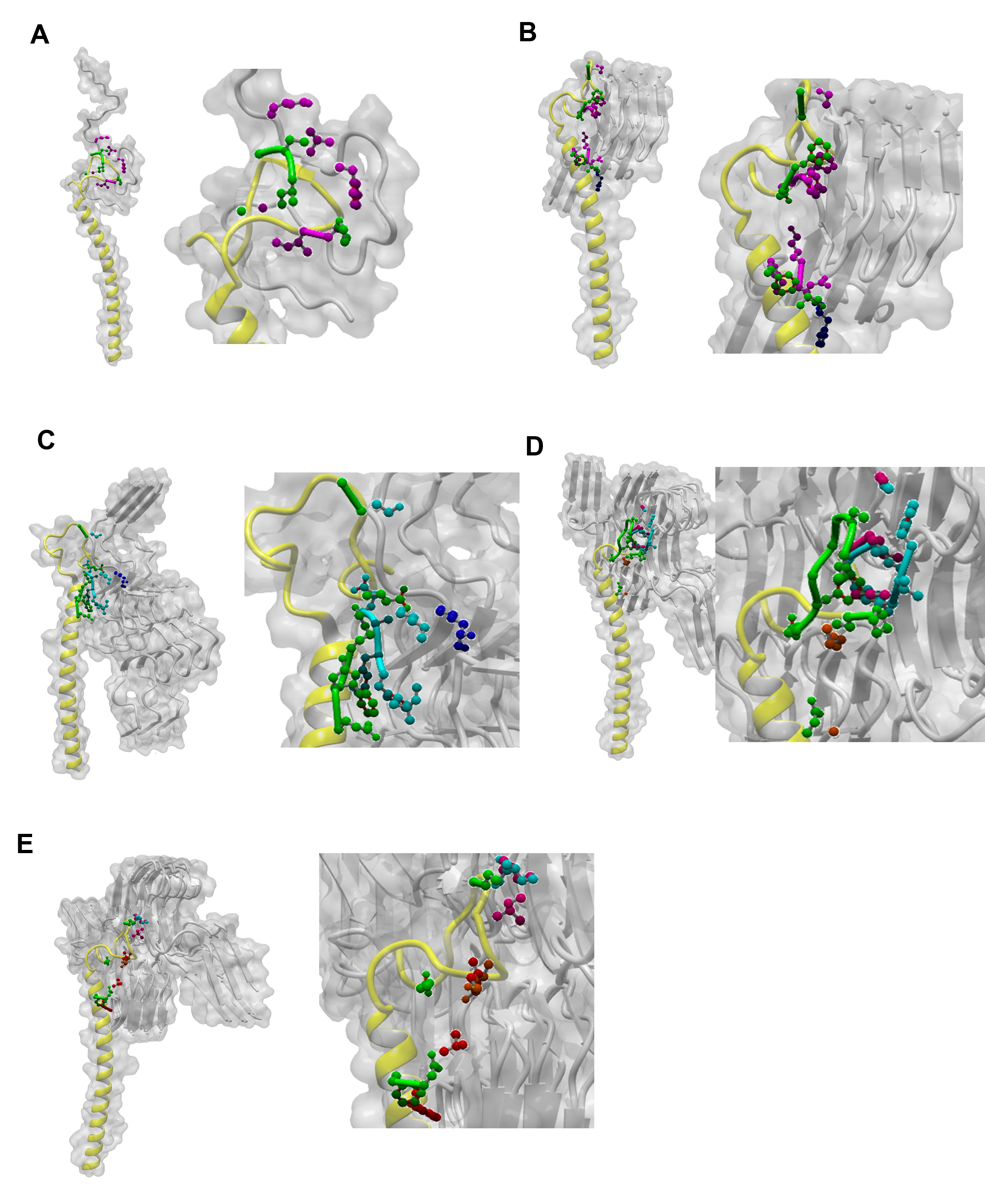

Supplement: SUPPLEMENTARY FIGURE S5 — Predictive interaction between Aβ and the 12CIII1 foreign peptide (Models 2). These images depict 3D docking models of different assembly states (1, 3, 6, 9, and 12) of Aβ (grey) interacting with the exposed region of 12CIII1-pVIII-protein (yellow), obtained using the ZDOCK server. Each structure is displayed in cartoon style, with amino acids involved in the interaction highlighted: residues from 12CIII1-pVIII-protein are shown in green, while those from Aβ are in various colors using the Ball & Stick style. [file Image_5.TIF]

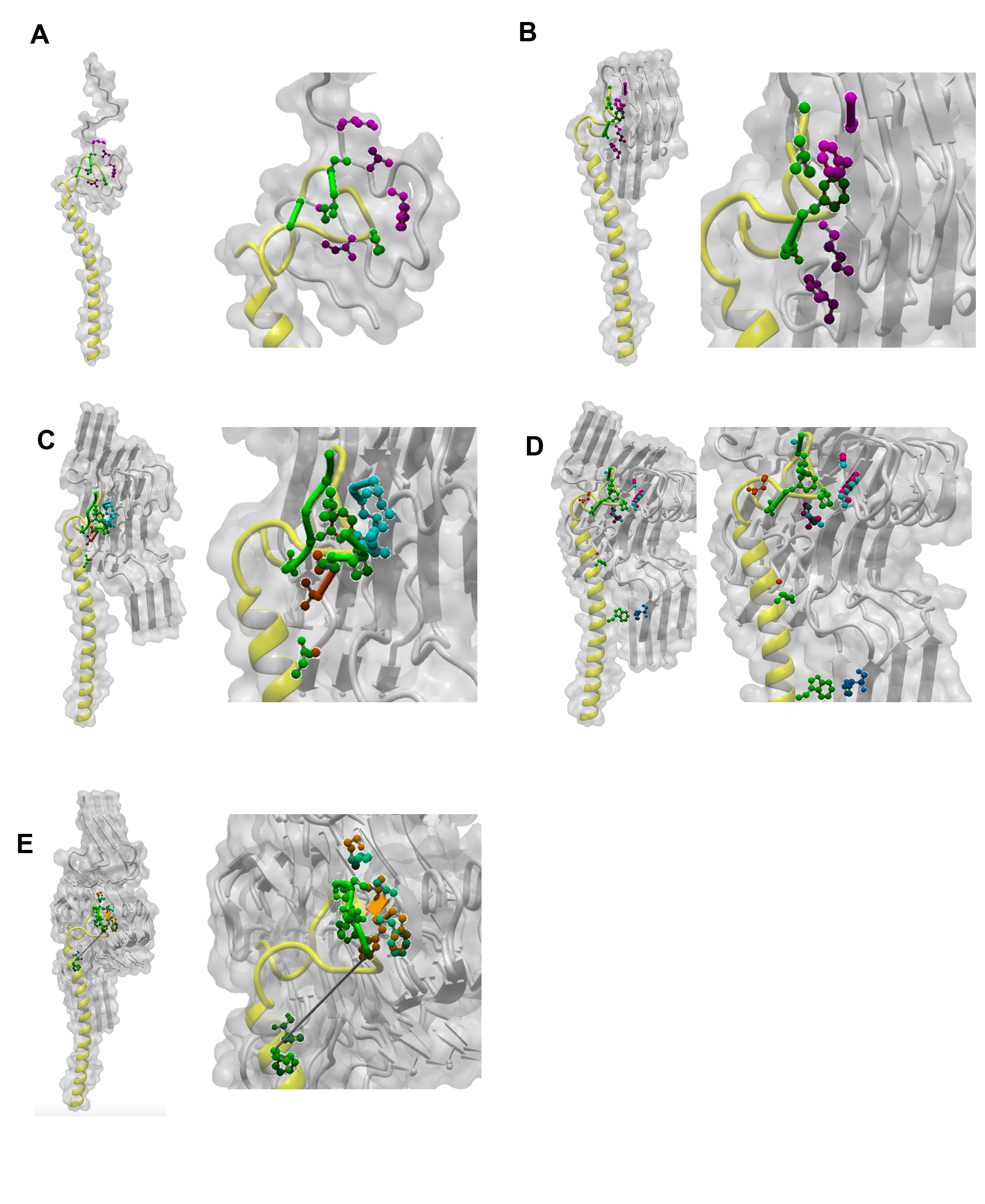

Supplement: SUPPLEMENTARY FIGURE S6 — Predictive interaction between Aβ and the 12CIII1 foreign peptide (Models 3). These images depict 3D docking models of different assembly states (1, 3, 6, 9, and 12) of Aβ (grey) interacting with the exposed region of 12CIII1-pVIII-protein (yellow), obtained using the ZDOCK server. Each structure is displayed in cartoon style, with amino acids involved in the interaction highlighted: residues from 12CIII1-pVIII-protein are shown in green, while those from Aβ are in various colors using the Ball & Stick style. [file Image_6.TIF]
