# Supplementary material for: A phage displaying an Aβ-interacting peptide mitigates neurotoxicity and prevents Aβ-driven gene expression changes
Source: Front Mol Neurosci. 2025 Dec 10;18:1716626. doi: 10.3389/fnmol.2025.1716626 (PMC12728043; doi:10.3389/fnmol.2025.1716626)
Supplement: Supplementary file 7 [file Table_1.DOCX]

**Supplementary Table S1. Scores of the analysis.** For each sample, the table shows the number of input reads after trimming (# of reads), the percentage of reads mapped against the reference genome (% mapped reads) and the mean depth of the covered regions (Mean depth).

| **Sample** | **# of reads** | **% mapped reads** | **Mean depth** |
| --- | --- | --- | --- |
| CTL-1 | 32289350 | 74.19 | 150.002 |
| CTL-2 | 25548914 | 67.70 | 103.829 |
| Aβ-1 | 12658906 | 58.72 | 40.1883 |
| Aβ-2 | 12487171 | 49.94 | 29.6092 |
| Aβ+12CIII-1 | 16974578 | 64.25 | 64.6914 |
| Aβ+12CIII-2 | 11007943 | 63.46 | 62.2212 |
